# Kinetic Proofreading Can Enhance Specificity in a Nonenzymatic DNA Strand Displacement Network

**DOI:** 10.1021/jacs.3c14673

**Published:** 2024-07-01

**Authors:** Rakesh Mukherjee, Aditya Sengar, Javier Cabello-García, Thomas E. Ouldridge

**Affiliations:** † Department of Bioengineering, Imperial College London, London SW7 2AZ, U.K.; ‡ Department of Chemistry, University College London, London WC1E 6BT, U.K.

## Abstract

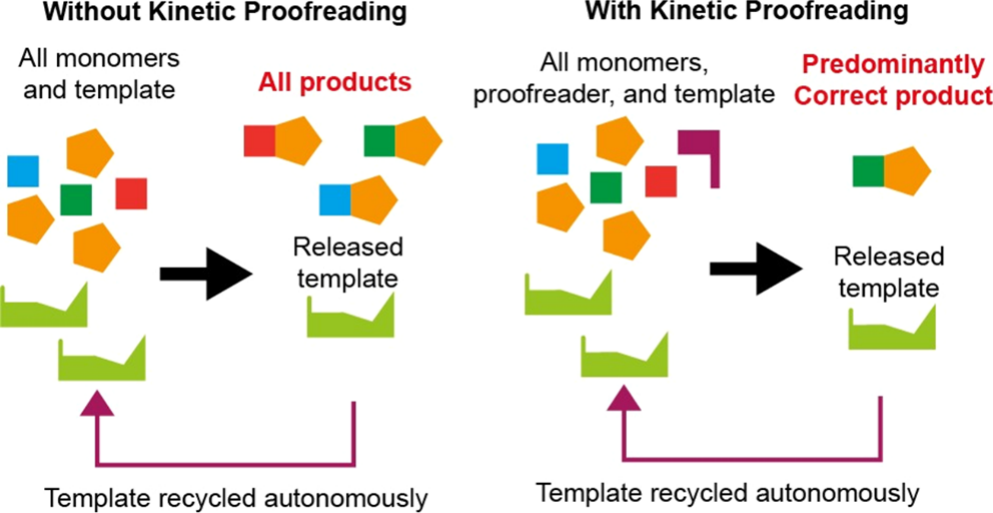

Kinetic proofreading is used throughout natural systems to enhance the specificity of molecular recognition. At its most basic level, kinetic proofreading uses a supply of chemical fuel to drive a recognition interaction out of equilibrium, allowing a single free-energy difference between correct and incorrect targets to be exploited two or more times. Despite its importance in biology, there has been little effort to incorporate kinetic proofreading into synthetic systems in which molecular recognition is important, such as nucleic acid nanotechnology. In this article, we introduce a DNA strand displacement-based kinetic proofreading motif, showing that the consumption of a DNA-based fuel can be used to enhance molecular recognition during a templated dimerization reaction. We then show that kinetic proofreading can enhance the specificity with which a probe discriminates single nucleotide mutations, both in terms of the initial rate with which the probe reacts and the long-time behavior.

## Introduction

**Figure 1:**
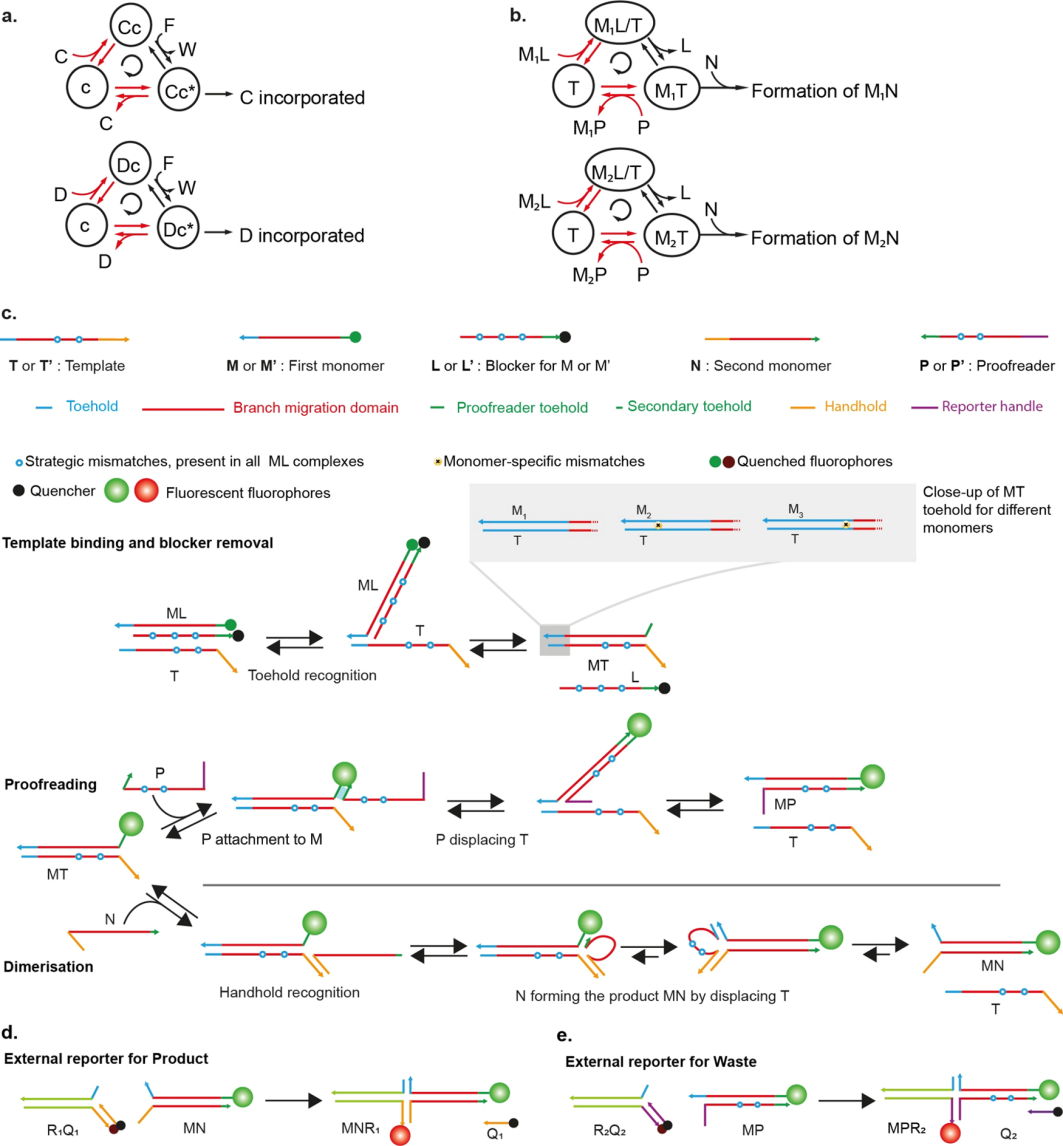
Kinetic proofreading networks. (a) Hopfield’s KP model. Recognition site c can bind to competing molecules C or D. Chemical free energy from the conversion of F(uel) into W(aste) drives the state of the recognition site clockwise around a cycle. The red-colored arrows show two stages at which discrimination between C and the weaker binding D is expected to occur, leading to a double enrichment of Cc* relative to Dc* and hence an enhanced relative rate at which C is incorporated into a product. (b) Proposed synthetic KP motif, highlighting its topological similarity to (a). The proposed system uses a template T to recognize and dimerize monomers M and N, selecting M_1_ from the competing variants. The monomer M is initially sequestered in a complex ML; conversion of this complex into MP releases the free energy to drive the template clockwise around the proofreading cycle, allowing discrimination at two stages (red arrows) to be manifest. (c) Domain-level design of a DNA-based kinetic proofreading network. The blocked monomer ML binds to the template T in two steps via TMSD to form the intermediate MT complex. The intermediate can be converted into MP waste complex by the proofreader, or dimerized via an HMSD reaction with the second monomer N to form the dimeric product MN. After either process, template T is released to act as a substrate in another reaction cycle. Variants M_2_ and M_3_ have a single nucleotide mismatch with the template toehold, which is used at two stages to provide discrimination (red arrows in (b)). Blue circles with white core indicate strategic mismatches that provide thermodynamic drive for all of the monomers. The number of such mismatches varies between different competing sets of monomer strands, as noted in the following sections, but remains the same within a set of competing monomers. The invasion of ML by T is reported by the increase in fluorescence of the monomer strands. Product and waste formation are reported by two external reporters (d, e). Further design details are given in Figures S1 and S2.

Specificity of molecular interactions is at the heart of biology. In particular, selective nucleotide base pairing drives information propagation in DNA replication, RNA transcription, and protein translation. Most simply, molecular recognition can be driven by differences in binding free energy between two candidate molecules and a substrate, resulting in an equilibrium with a bias toward one candidate. However, in many biosynthetic processes, the specificity of correct product formation is orders of magnitude higher than that suggested by free-energy differences, despite additional complications that make discrimination more challenging than implied by the equilibrium picture.^1^ Translation of protein from mRNA operates with an error rate^2,3^ of 10^–4^ and DNA replication^4^ with an astonishingly low error rate of 10^–9^. To describe such unusually high accuracy, Hopfield^5^ and Ninio^6^ independently proposed “kinetic proofreading” (KP) (Figure 1a), in which small free-energy differences are utilized repeatedly over multiple steps in a reaction cycle to achieve a significant overall discrimination. The idea has been widely applied, and the same basic principle has been identified in antigen recognition by T-cells,^7^ aminoacylation of tRNA,^8^ disentanglement of DNA by topoisomerases,^9^ specific protein degradation via ubiquitination,^10^ and chromatin remodeling.^11^

Hopfield’s KP is illustrated in Figure 1a. Hopfield envisioned a single recognition site c discriminating between two candidate molecules C (correct) and D (incorrect) for incorporation into a product. Either molecule can bind to c, forming Cc or Dc. Then, the molecule can either unbind or the complex undergoes a chemical transition to another bound state, Cc* or Dc*. This moment is the first discrimination point: D will have less affinity for c than C and will unbind faster; D is thus less likely to proceed to the second bound state. From the second bound state, there is another opportunity to detach prior to incorporation into the final product, giving a second opportunity to discriminate. The two stages of discrimination can doubly enrich the correct product over the incorrect product.

Central to Hopfield’s KP is the driving of a single recognition site c through a cycle of states (Figure 1a).^12^ Chemical free energy must be consumed to ensure systematic motion around the cycle. The result is that the Cc*/Dc* ratio can be maintained out of equilibrium, which allows it to be doubly sensitive to the difference in binding energy between the two candidates.

Discriminating similar molecules is also a ubiquitous motif in synthetic nanotechnology, whether in computational strand displacement cascades,^13^ tile assembly systems,^14^ or diagnostics-based applications.^15^ The ability to discriminate between perfectly matching and almost matching sequences is particularly relevant to single nucleotide polymorphism (SNP) detection. SNPs are single nucleotide mutations in specific gene sequences and are markers for various diseases.^16^

Nanotechnologists have designed elegant synthetic DNA nanotechnology-based systems in which discrimination between intended and unintended complexes is enhanced relative to a baseline.^17−21^ Typically, the approach is to modify a design to increase the number of mismatched base pairs that must be formed to make the unintended product. Although such an approach, which may not always be possible in complex networks, implements a form of proofreading, it does not constitute nonequilibrium KP, where a single free-energy difference is exploited multiple times. Indeed, to the best of our knowledge, *de novo* KP has not previously been demonstrated in synthetic systems, despite its importance in nature. This fact suggests a fundamental limitation in both our understanding of and ability to design biochemical networks.

In this article, we demonstrate that KP can be implemented in nonenzymatic, DNA-based systems to improve discrimination between very similar recognition domains through a fuel-consuming cycle. Specifically, we apply KP to enhance the specificity with which a molecule is incorporated into a two-stranded DNA dimer by a catalytic template, a synthetic analog of tRNA charging. We first characterize the individual discrimination steps and then demonstrate that the proofreading motif enhances the overall specificity of dimerization. Finally, we demonstrate the utility of KP by showing that it can enhance a simple network for the detection of generic SNPs in ssDNA.

In so doing, we (a) explore a fundamental biochemical motif by engineering a minimal example *de novo* and (b) demonstrate that it is possible to implement KP in the specific context of DNA strand displacement networks. For the former motivation, constructing a minimal synthetic KP motif tests our understanding of the fundamental biochemistry, potentially revealing subtleties that are absent in idealized models. For the latter, we open the door to the application of KP as a general mechanism to a range of DNA strand displacement-based systems^22^ that need to distinguish between correct and incorrect products with similar free energies. Since KP is a way of improving the specificity of a recognition process, it can be applied even to systems that already discriminate quite well, allowing the intended targets to be distinguished from even larger pools of competing molecules.

## Materials and Methods

### Sequence Design and Nomenclature

All sequences were generated using the NUPACK web server to minimize undesired secondary structures. Some strand designs were manually altered to incorporate strategic features such as mismatches. All of the strands were purchased from Integrated DNA Technologies as HPLC purified at 100 μM concentrations in IDTE buffer, pH 8. All of the sequences used in the study are listed in Tables S15–S17. Detailed design considerations are shown in Figures S1 and S2.

The first type of monomers, named M_1_, M_2_, and M_3_, are used for characterizing monomer binding, sequence-specific proofreading, and complete discard mechanism. M_1_ is the “correct” monomer, and M_2_ and M_3_ are the “incorrect” strands. The blocker strand L is used for the monomer-blocker duplex. P_6_, P_7_, and P_8_ are the proofreader strands used for the set of monomers. An altered design of the first type of monomers, M′_1_, M′_2_, and M′_3_, was used for the final templated dimer formation along with the second type of monomer N. Similarly, altered template T’, blocker L′, and proofreaders P′_6_, P′_7_, and P′_8_ were used with the second set of monomers. Generic terms M, P, T, and R were used to denote all strands belonging to one of these categories.

External fluorescence reporters, denoted generically as RQ, as shown in Figures 1d,e and 5a, are formed by three strands. The strand bearing the quencher species is denoted as Q; the other two partially complementary strands are collectively denoted as R. R_1_Q_1_ is the reporter for the dimeric product M’N and R_2_Q_2_ for the MP wastes.

### General Annealing Protocol

The ML and M′L′ duplexes were prepared by annealing 100 nM M or M′ with 20% excess L or L′ to ensure that all M or M′ strands were bound to L strands. To prepare MT and MP complexes, 100 nM M strands were annealed with 20% excess T or P strands. Reporter complexes RQ comprised of three strands, Rcomp, *r*_*i*–*j*_ (collectively called R), and Q (details in Figure S2 and Tables S15–S17). For this, 200 nM Rcomp strands were annealed with 20% excess of *r*_*i*–*j*_ and Q strands. The volumes of the annealing mixtures were 100 or 200 μL depending on the experimental requirements. The required strands were mixed to have the final concentrations in 1× Tris-Acetate-EDTA buffer containing 1 M NaCl. This mixture was first heated to 95 °C and held there for 5 min, and then gradually cooled down to 20 °C at 1 °C/min and stored at 4 °C until used.

### Fluorescence Kinetics Measurements and Calibration

All fluorescence measurements were performed in a BMG Labtech CLARIOstar fluorescence plate reader in Greiner μClear flat-bottomed 96-well plates. Reactions were initiated, unless otherwise stated, by injecting 50 μL of trigger (usually a combination of single-stranded or duplexed DNA oligonucleotides with buffer) into 150 μL of the other reactants using the instrument’s in-built injectors (pump speed 430 μL/s). The final mixtures were then shaken for 10–30 s at 400–500 rpm before measuring the fluorescence.

Monomers M_1_, M_2_, M_3_, M′_1_, M′_2_, M′_3_, and Rsnp were labeled with fluorophore Cy3. L, L′, and Qsnp were labeled with IowaBlack FQ quencher. Rcomp-AF and RcompP were labeled with AlexaFluor-647. Q_1_ and Q_2_ were labeled with IowaBlack RQ quencher.

For the Cy3 and AlexaFluor-647 measurements, presets from the instruments were used for excitation and emission wavelengths. Specifically, for Cy3, excitation: 530–20 nm, emission: 580–30 nm, gain: 2100; and for AlexaFluor-647, excitation: 625–30 nm, emission: 680–30 nm, gain: 2700. For each well, each measurement was taken as 20 flashes in a spiral scan with a maximum diameter of 4 mm. Step-by-step experimental methods used in each experiment are described in Supplementary Notes 1–8.

Fluorescence calibrations were performed for fluorophore-labeled complexes to quantify the fluorescence signals obtained from unit concentration of such complexes. Cy3 (for ML, MP, MT complexes) and AlexaFluor-647 (for RQ and MPR complexes) signals were monitored in a volume of 200 μL with different candidates’ concentrations ranging from 0 to 20 nM in 1× TAE buffer with 1 M NaCl. Only M_1_ and P_6_ were used rather than testing all possible permutations of monomers and proofreaders because the local environments of the fluorophores in all complexes were the same.

### Data Processing and Analysis

This section describes the methods used for the estimation of the reaction rate constants for various intermediary reactions. All rate constants are obtained from the experimental reaction data set as obtained from the Cy3 and AlexaFluor-647 measurements shown in Figures S16, S19, S22, and S26. The AlexaFluor-647 channel gives a fluorescent signal in the presence of RQ, MPR, and PR, whereas Cy3 gives a signal for ML, MT, MP, and MPR.

### Trajectory Analysis and Rate Constant Estimation

We used the ParametricNDSolve built-in function in Mathematica 12.3 to fit mass action models of reaction kinetics to the processed data. The ODE models used to describe each experiment are given in Supplementary Notes 10–13. In general, the procedure involved fitting a single set of rate constants to a number of kinetic traces under distinct initial conditions. In each case, we defined an array *K*_n_ of known rates (determined by earlier fits to different experiments) and an array *K*_un_ of unknown rates. The system of ODEs was constructed within the ParametricNDSolve function, and all of the starting conditions, initial concentration values and *K*_n_, were provided. ParametricNDSolve was then used to minimize the mean-squared error (MSE)
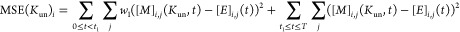
1over possible values of *K*_un_. Here, [*M*]_*i*,*j*_(*K*_un_,*t*) is the predicted concentration of the chosen species *i* in kinetic trace *j* at time *T*, given *K*_un_ as the values of the unknown rate constants. Concurrently, [*E*]_*i*,*j*_(*t*) corresponds to the data observed in the experiment. To improve the accuracy with which fits captured the behavior at early time points, weights *w*_1_ = 1 and *w*_2_ = 0.2 were assigned to data points with *t* < *t*_1_ and *t*_1_ ≤ *t*, respectively. *t*_1_ is approximately equal to the time instance when the species concentration has plateaued and was manually defined for each reaction. Each element in the set *K*_*un*_ is sampled from a variable range of values that can be found in Supplementary Notes 10–13.

The data on the binding of blocked monomers to templates were used for identifying rate constants governing the displacement of blockers from monomers and vice versa. The procedure is outlined in Supplementary Note 10. The fitted parameters can be found in Table S9, and the comparison between experimental observations and simulation predictions is shown in Figure S32. Subsequently, data pertaining to the triggering of reporter complexes by pre-prepared MP duplexes were utilized to determine rate constants for the reporter, as explained in Supplementary Note 11. Owing to the challenges in unequivocally determining the total monomer concentration in these reactions, eight distinct sets of rate constants for each reporter were generated, based on plausible estimates of the total monomer concentration. These eight sets of reporter rate constants, in conjunction with the rate constants for template binding, were employed in a simultaneous fit of the template recovery and full discard pathway experiments. The set of rate constants that minimized the total error for template recovery and discard pathway was then selected. The procedure is outlined in more detail in Supplementary Note 11. The resulting fits for reporter characterization are shown in Figures S34–S36; the fits for template recovery are shown in Figures S37–S39; and the fits for discard pathway are shown in Figures S40–S42. The fitted rate constants are presented in Tables S10 and S11.

### Model-Based Predictions for Intermediates and Dimerization

The values of rate constants obtained from fits were used to predict the time-varying concentration of MT complexes during the full discard pathway experiments (Figure S43). The predictions were made by numerically integrating eqs S35–S39 using the rate constants in Tables S9–S11 and the experimental initial conditions as parameters.

Although the dimerization system used redesigned monomers and templates, we nonetheless used the rate constants obtained from initial fits to predict the yield of dimers over time. The predictions were made by integrating eqs S51–S53 using the rate constants in Tables S9–S11 and the experimental initial conditions as parameters. A plausible dimerization rate constant of 3.50 × 10^05^ M^–1^ s^–1^ was also used. The results are plotted in Figure S53. At variance with the experimental results, the model predicts a high dimerization yield for all monomers and proofreaders in the long-time limit. We suspect that this prediction is due to an excessive reverse proofreading (rebinding of proofread monomers to the template) rate constant for mismatched monomers; these constants are not well constrained by the data.

## Results

### Network Design for Kinetic Proofreading

We construct an enzyme-free, DNA-based synthetic KP system. DNA is used purely as an engineerable molecule that predictably assembles into well-defined structures driven by Watson–Crick–Franklin base pairing. Our KP system exploits the widely used motif of toehold-mediated strand displacement^22−25^ (TMSD). In TMSD, a single invader strand displaces an incumbent from an incumbent-target duplex (see Figure 1c). The process is accelerated and pushed thermodynamically downhill by a toehold of available bases in the target to which only the invader can bind. Alongside TMSD, we also exploit the handhold-mediated strand displacement^26−28^ (HMSD) reaction (also shown in Figure 1c). HMSD is analogous to TMSD, but the binding to the target is accelerated by a handhold in the incumbent rather than the target.

The network is illustrated at a domain level in Figure 1c. Domains are sections of single-stranded (ss)DNA that are designed to bind as a collective unit. In this network, the monomers M and N that form MN are each single strands of DNA (rather than individual nucleotides or amino acids). Dimerization in solution is inhibited by the presence of a blocker strand L bound to M but can occur rapidly via a template T, which acts as the substrate in our system. First, using a toehold (t_T_), M can bind to T via TMSD and remove L, revealing a second short toehold domain (t_P_).^29^ The newly available toehold t_P_ can be used by the proofreader strand P to initiate a second TMSD reaction to form a waste duplex W, or a second monomer N can bind to the handhold h and then complete HMSD to form a dimeric product MN. Effectively, the template T catalyzes two competing noncovalent processes: a two-step toehold exchange^30−32^ in which ML + P is converted into MP + L, and an HMSD-mediated dimerization^26−28^ with ML + N being converted to MN + L.

From the perspective of the template, the process is a direct analog of Hopfield’s KP (compare Figure 1a,b). The free energy released by converting ML + P into MP + L drives the template round the sequence of states T → ML/T → MT → T, with conversion to the final product MN happening from the MT state. This nonequilibrium cycling allows for the same difference in binding free energy between three versions of the first monomer: M_1_, M_2_, and M_3_, to be exploited at two stages. These monomers differ by a single base in the toehold recognition domain t_T_; M_1_ has a perfectly matching toehold for template recognition, whereas M_2_ and M_3_ have one mismatch at different positions in the toehold. First, after the initial binding of M to T, the strength of the toehold t_T_ determines the probability that the monomer will remain bound for long enough for blocker displacement.
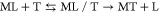
2

Second, a stronger toehold interaction can also reduce the probability of successful displacement by the proofreader,^33^
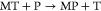
3allowing for the concentration of M_1_T to be further enriched relative to M_2_T or M_3_T, thereby enhancing the relative rate at which M_1_N is formed.

Although the synthetic KP motif is equivalent to Hopfield’s KP motif from the perspective of the template, there is an important difference. In our design, the thermodynamic drive pushing T around the proofreading loop comes from converting the monomers themselves into waste, rather than ancillary fuel molecules. We will return to the consequences of this difference in the conclusion.

Complete design details, including sequences, are provided in Figure S1 and Tables S15 and S16. Long binding domains (red domain, Figure 1c) contain mismatched base pairs (blue circles) in the initial ML complexes; these mismatches are gradually eliminated during subsequent reactions. The mismatches are common to all monomers and provide a “hidden thermodynamic drive”^33^ toward formation of the final product MN.

We initially consider a fixed set of monomer, template, and blocker strands. We explore the performance of the individual substeps and the overall network for different designs of the proofreader strand.

### Monomers Binding to the Template

**Figure 2:**
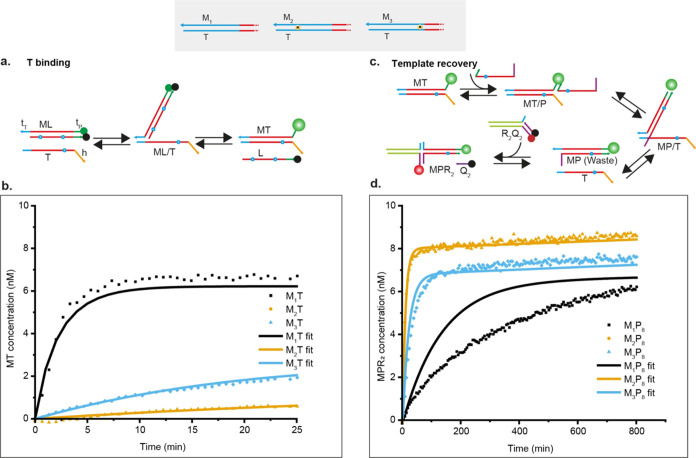
Two stages of discrimination in the KP experiment. (a) Schematic of template binding showing that template T invades the blocked monomer ML by TMSD to form MT. (b) Kinetics of the template binding process obtained from the Cy3 signal of the M strand. 8 nM M_1_L, M_2_L, and M_3_L were added in separate wells, and the reactions were initiated by injecting 10 nM T in each well. Obtained fluorescence signals are converted into concentrations using calibration curves of known concentrations (Supplementary Note 11). Solid lines are fits to an ODE model fitted to several experimental conditions simultaneously. (c) Template recovery is the removal of M from T by the proofreader molecule P. Formed waste MP is reported by the external reporter R_2_Q_2_, which is labeled with AlexaFluor-647. (d) Kinetics of the template recovery step were obtained from the AlexaFluor-647 signals. 8 nM M_1_T, M_2_T, and M_3_T and 20 nM of their respective reporter complexes were added in separate wells. 50 nM P_8_ was added to each well to trigger the reaction. Solid lines are fits to an ODE model (Supplementary Note 12) fitted to several experimental conditions simultaneously (Figures S37–S39).

First, we consider the rates of invasion of blocked monomer complexes ML by the template T. The monomers were labeled with Cy3 fluorophore, and the blocker strand L was labeled with quencher IowaBlack FQ. The progress of the reaction was monitored by the increase in Cy3 fluorescence when L was displaced by T (Figure 2a). We performed the reaction with a range of T concentrations for 8 nM M_1_, M_2_ and M_3_; the results for [T] = 10 nM are plotted in Figure 2b, showing the correct monomer M_1_ forming a duplex with T faster and with a higher equilibrium yield^15^ than the mismatched monomers (Figure S32). Solid lines in Figure 2b show fits to an ODE model for each system (Supplementary Note 11); these fits used a single set of parameters for each system to fit all concentrations of T.

### Sequence-Specific Proofreading

As a second step, we looked at the efficacy and sequence specificity with which proofreader strands P remove monomers from the template, allowing “template recovery” to participate in another reaction. We explored three different designs for P; each had the same 5-base toehold t_P_, but we modified the thermodynamic strength of the MP waste product by truncating the displacement domain of P (shown in red in Figure 2c). We label the designs P_6_, P_7_, and P_8_, with 6, 7, and 8 being the number of bases in the toehold and displacement domain of M that are left unpaired after the proofreading reaction. P_6_ thus represents the most thermodynamically favored proofreader and P_8_ the least. In all cases, reactions were driven by adding a large excess of P, which constitutes a fuel reservoir. For all combinations of proofreaders and monomers, a range of concentrations of MT complexes were added to a large excess of proofreaders; the formation of M_1_P waste was reported by an external reporter R_2_Q_2_, which was labeled with AlexaFluor-647–IowaBlack RQ fluorophore–quencher pair and was already present in solution (Figures 2c and S34–S36 and Supplementary Note 4). The results for MT = 8 nM and P_8_ are shown in Figure 2d; full results are shown in Figures S37–S39.

Again, we also report fits to an ODE model for each system; these fits used a single set of parameters for each proofreader, fitted simultaneously to all template recovery experiments as well as subsequent experiments on the full proofreading cycle. As expected, P_6_, which had the strongest binding to M, was the fastest in forming the waste duplex PM. Other proofreaders were slower but still demonstrated template recovery. Crucially, P_7_ and P_8_ showed clear discrimination between M_1_, M_2_, and M_3_, with M_1_ being removed from T much more slowly, as evident for P_8_ in Figure 2d and borne out by the fitted rate constants (Table S11). Alongside the discrimination present in the initial template binding, the existence of this second discrimination step demonstrates the potential for kinetic proofreading. For P_6_, removal by proofreader appears slightly slower for M_1_ than M_2_ and M_3_, but the distinction is smaller, and the relatively slow reporter rate complicates the analysis.

The action of the proofreaders is fundamentally different from the presence of a large reservoir of blocker strands. Adding a large concentration of blocker strands to MT complexes does result in the removal of M and the recovery of T, as shown outlined in Supplementary Note 2 and Figure S33. However, removal of M_2_ and M_3_ is not substantially faster than M_1._ The relative rates for template displacing blocker and blocker displacing template are fundamentally constrained by the free-energy change of the process. The overall discrimination is equilibrium in nature, and if the full equilibrium discrimination is manifested in the rate of template binding, no discrimination will be observed in the reverse reaction.^34^ In contrast, using a distinct proofreader strand allows, in principle, the initial template binding and subsequent template recovery steps to be thermodynamically decoupled by fuel consumption. In practice, we rely on a single additional mismatch between blocker and template to manifest this difference; previous work has shown that a well-placed single mismatch can have large kinetic and thermodynamic effects.

### Complete Discard Mechanism

**Figure 3:**
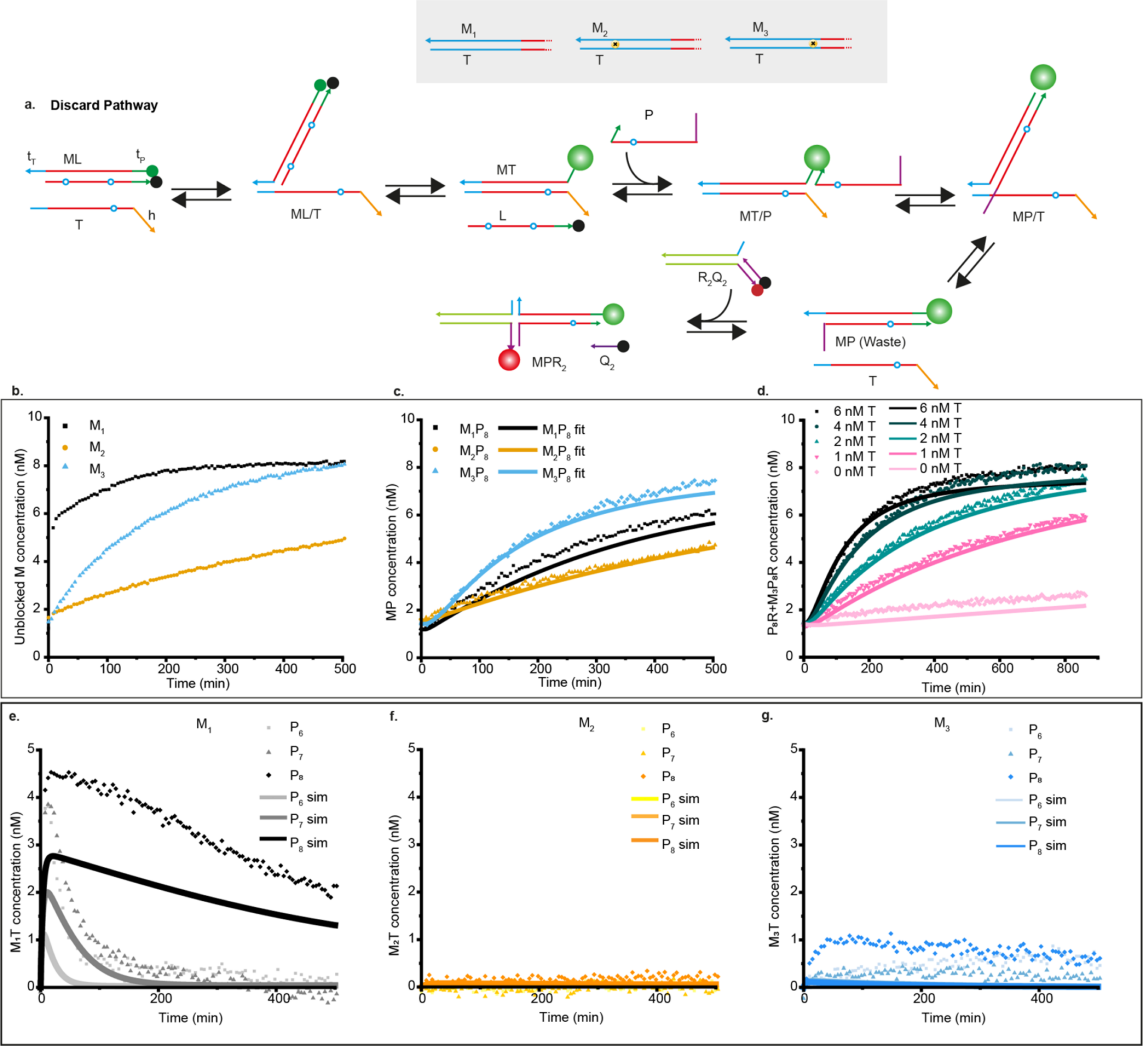
Kinetics of the full discard pathway. (a) The full discard pathway starts with the blocked monomer, proofreader, and reporter and is triggered by the injection of T, which displaces the blocker from M and is subsequently displaced itself by the proofreader strand. (b) Concentration of M liberated from its blocker after injection of 4 nM T, as inferred from Cy3 fluorescence, for 8 nM M_1_L, M_2_L, and M_3_L in different wells, each with 50 nM P_8_ and 20 nM of their corresponding reporters. (c) Formation of MP waste in the same reactions, as inferred from reporter fluorescence. Solid lines are fits to an ODE model fitted to several experimental conditions simultaneously (Supplementary Note 13). (d) Fluorescence of reporter for M_3_P_8_ waste after triggering a mixture of M_3_L and P_8_ with different concentrations of T. Rates of waste formation increased with increased concentrations of T. For all cases, formed waste concentrations are higher than the added template which indicates repeated catalytic activity. (e) Concentration of intermediate M_1_T over time for three different proofreaders, P_6_, P_7_, and P_8_. Concentration of M_1_T was estimated by subtracting AlexaFluor-647 signals from Cy3 signals converted to concentration. (f, g) Concentration of M_2_T and M_3_T over time in equivalent experiments. Solid lines in (e–g) are estimates of MT from the fitted ODE model.

We now analyze the entire catalytic discard mechanism in which the blocked monomers first bind to the template to form MT duplexes and are subsequently converted into waste complexes by P (Figure 3a). We added T to solutions of M_1_, M_2_ or M_3_, P_6_, P_7_ or P_8_, and the appropriate waste reporter and tracked both the amount of monomers that had been liberated from blocker strands and the amount of waste in solution. Results for P_8_ and T = 4 nM are shown in Figure 3b,c, and full results are shown in Figures S40–S42. Fits of the data to an ODE model were performed simultaneously with the data on template recovery, using a single set of parameters for each proofreader (Supplementary Note 13).

The speed of MP complex formation depends on both the rate of initial template binding and proofreader-driven template recovery. The first step is faster for M_1_, and the second step is faster for M_2_ and M_3_. More importantly, the system exhibits clear catalytic turnover as multiple monomers can be converted into waste for each template, with systems at low template concentrations producing output signals several times the initial template concentration (Figures 3d and S40–S42). Proofreading thus returns a functional template as required.

The purpose of KP is to enrich the population of template-bound M_1_ monomers relative to template-bound M_2_ and M_3_. The concentrations of each template-bound monomer can be estimated by subtracting the waste concentration from the unblocked monomer concentration (subtracting Figure 3c from b for P_8_ and T = 4 nM). Although this approach is crude, it provides clear evidence for a spike in M_1_T at short times, with M_2_T and M_3_T highly suppressed at all time points (Figures 3d–f and S43). This behavior is qualitatively consistent with predictions of an ODE model, parametrized by fits to earlier experiments (Figure S43). We suspect that the apparent ∼1 nM yield of M_3_T at long times is likely an artifact of the crude methodology. This interpretation is supported by the fact that the apparent yield of M_3_T is almost as high for P_6_ as P_8_ despite P_6_ having much higher affinity for M_3_.

### Templated Dimer Formation with Kinetic Proofreading

Having discriminated between the correct and incorrect monomers at both the template binding and template recovery steps, we redesigned the DNA strands to allow for dimerization through HMSD, using a handhold domain in a new template, T′. We also redesigned the monomers, now called M′_1_, M′_2_, and M′_3_, to accommodate three mismatches between the blocker and monomers (1Figure 1c), rather than two. One of those mismatches is eliminated in template binding and the remaining two during HMSD. In this redesign, we retained the domain lengths from the previous characterization process but changed the sequences (Tables S15–S17).

The handhold recognizes the second monomer N, allowing dimerization, via HMSD (Figure 1c). The intermediate M′T′ can also be invaded by the redesigned proofreader P' to form waste M′P′, allowing kinetic proofreading of M′. We explored this process by adding various template concentrations to mixtures of monomers and proofreaders, with M′_1_, M′_2_, and M′_3_ probed separately. Dimer concentration was reported by an external reporter similar to that used for waste characterization (Figure 1d). We observed that the reporter had a small but significant tendency to react directly with the monomers (Figure S44). We therefore monitored lock strand removal by the increase in Cy3 signal intensity and added the external reporter only after the Cy3 signal reached a plateau for all template-containing systems.

**Figure 4:**
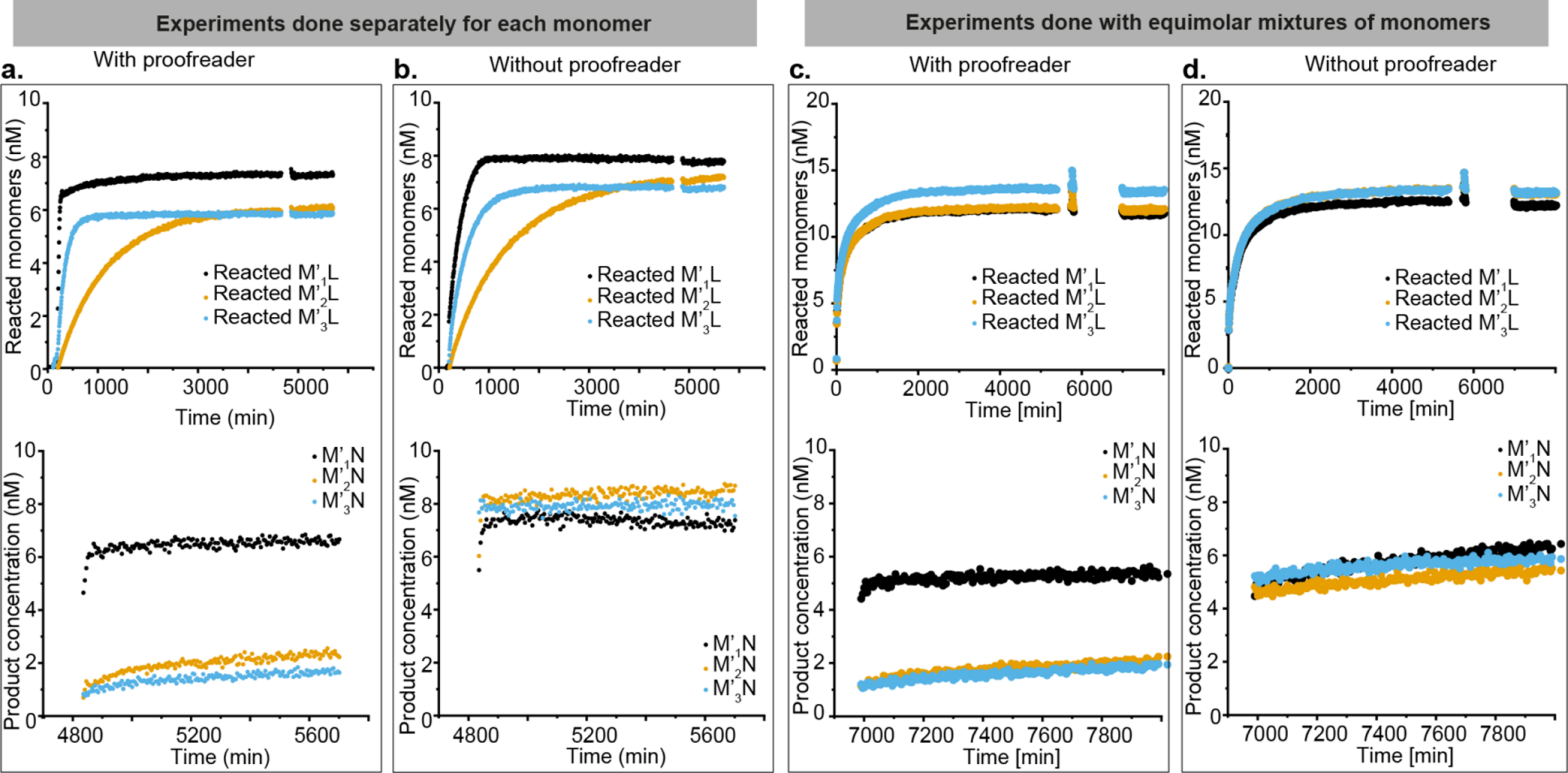
Kinetic proofreading in a dimerization process. (a) Top: Concentration of reacted monomers in a dimerization reaction with proofreaders. 8 nM blocked monomers M’_1_, M’_2_ and M’_3_ in separate wells were mixed with 10 nM N, 50 nM P’_6_, and the reactions were triggered by injecting 2 nM T’. Unblocked monomer concentrations were inferred from Cy3 fluorescence. Bottom: Dimeric product formation by HMSD in the same reaction. After 3 days, the extent of product formation was assayed by adding 20 nM of the corresponding reporters; product concentration is inferred from reporter fluorescence, as outlined in Supplementary Note 15 and Figures S44–S49. We observe a much higher yield of M’_1_N than for the alternatives. (b) Top: Blocker strand removal from identical experiments in the absence of the proofreader. Bottom: Dimeric product formation in reactions without a proofreader is almost equal for all monomers. (c) Top: Total concentration of monomers liberated in a competitive system initiated with 5 nM each of M’_1_L’, M’_2_L’, and M’_3_L’, 15 nM N and 50 nM P’_6_ triggered with 2 nM T’. Three replicas were analyzed using the same approach as in (a), one to track each of M’_1_, M’_2_, and M’_3_ separately. Bottom: Product formation in these experiments. As in (a), we observe a much higher yield of M’_1_N than for the alternatives. (d) Top: Total concentration of monomers liberated in identical experiments to (c) but without P’_6_. Bottom: All products M’_1_N, M’_2_N, or M’_3_N have a high yield.

We tested the three variants P′_6_, P′_7_, and P′_8_, with the results for P′_6_ and 2 nM T shown in Figure 4a,b and the others in Figures S44–S52. All systems showed discrimination at the first step, which is not proofreader-dependent; the formation of M′_1_T′ is substantially faster than M′_2_T′ or M′_3_T′, as evidenced by the Cy3 signal. In the absence of the proofreader, however, systems with M′_1_, M′_2_, or M′_3_ all reach high concentrations of M′N eventually, as evidenced by the dimer reporter signal. By contrast, in the presence of proofreaders P′_6_ or P′_7_, the eventual production of M′_2_T′ and M′_3_T′ is strongly suppressed; the level of incorrect product in these cases is comparable to the inherent leak of the reporters (Figures S44–S52). Quantitatively, one may consider the discrimination factor D_*x*:*y*_, which in this case is given by the yield of M′*_x_*T′ relative to M′_y_T′ at the end of the experiments, since the total input concentration of each monomer is equal. In the absence of any proofreader, the discrimination factor is approximately one, because all monomers obtain essentially the same final yield. Specifically, for the data plotted in Figure 4b, D_1:2_ = 0.87 and D_1:3_ = 0.92 with no proofreader present. In the presence of P′_6_ (Figure 4a), we obtain D_1:2_ = 3.00 and D_1:3_ = 4.20, a 4–5-fold improvement. For the equivalent data for P′_7_, shown in Supplementary Note 18 and Table S12, we obtain D_1:2_ = 2.71 and D_1:3_ = 3.91.

This performance indicates successful proofreading, in which the alternative proofreading discard pathway competes effectively with incorporation into the dimerized state—although we note that the apparent mechanism for P′_6_ was a little unexpected (Supplementary Note 9). By contrast, P′_8_ does not show effective proofreading. We believe that, in this case, the proofreading pathway is too slow, allowing M′_2_T′ and M′_3_T′ to form even when P′_8_ is present. Model predictions based on the experiments in Figure 2 (Figure S53) show high yields of M’_2_T’ for P’_7_ and P′_8_; in the case of P′_8_, slower proofreading contributes to this response.

As with template recovery, the reservoir of proofreading molecules acts in a fundamentally different manner to an excess of blocker strands. In Figure S54, we show that an excess of blockers does not prevent the eventual formation of M′_2_T′ and M′_3_T′, and only has a weak effect on kinetics. To demonstrate that our KP network enhances discrimination from a mixed pool of distinct monomers, we prepared an equimolar mixture of M′_1_L, M′_2_L, and M′_3_L with an equal concentration of each duplex and an excess of N. As in the individual experiments, P′_6_ and P′_7_ again significantly suppressed the formation of incorrect dimers M′_2_N and M′_3_N while allowing M′_1_N to be formed at a high level (Figures 4c,d and S55–S60).

### SNP Identification via Kinetic Proofreading

**Figure 5:**
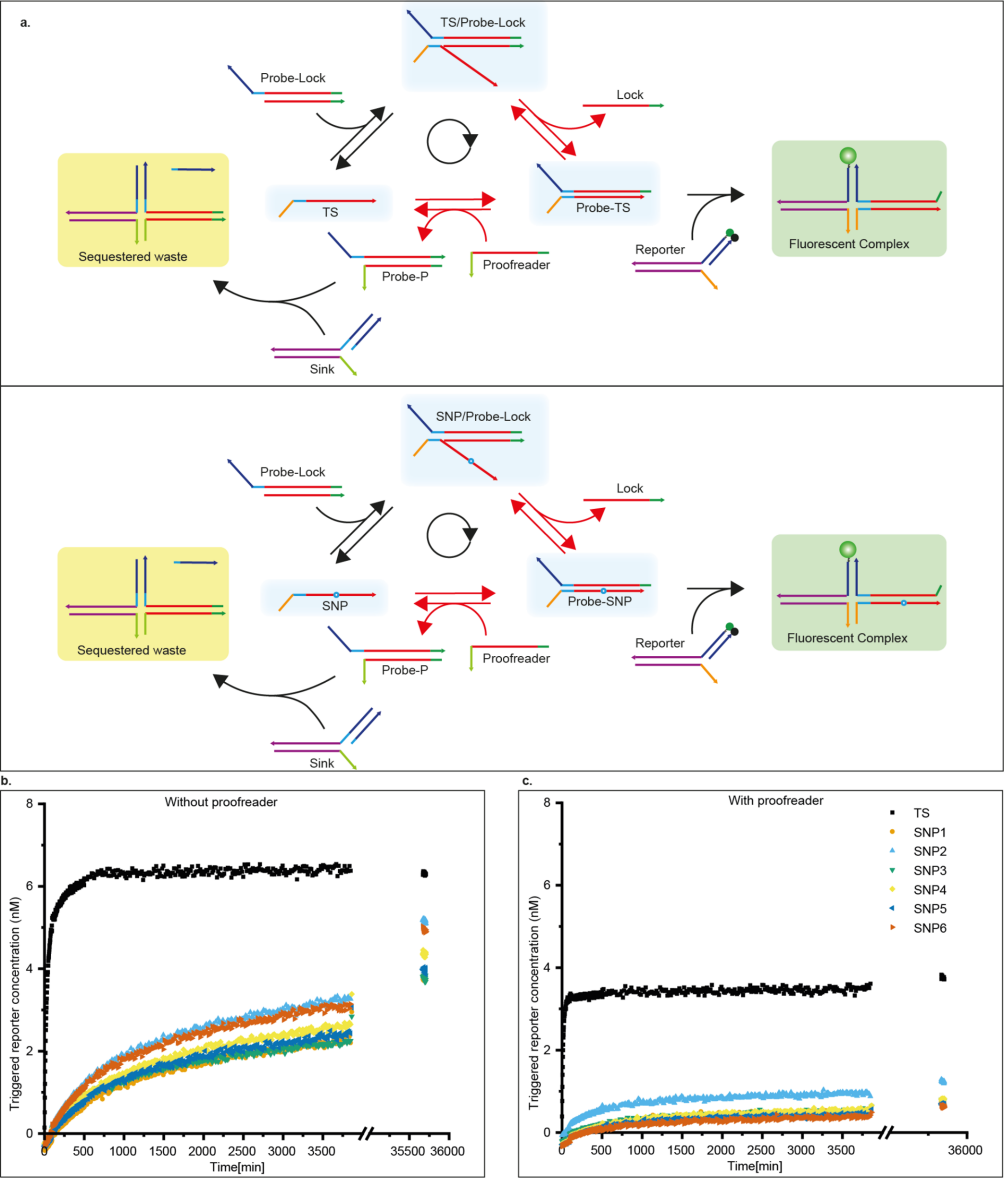
SNP identification scheme utilizing KP to suppress the output of the mismatched target. (a) Reaction scheme: TS and SNP strands bind to the blocked probe to form the intermediates TS-Probe and SNP-Probe, which trigger the fluorescence by displacing the quencher strands from the external reporters. Proofreader molecules can also invade these intermediates to convert the Probe into Waste, which is in turn sequestered into an inert complex by the Sink. The red-colored arrows denote two stages of discrimination between TS and SNP. (b) Fluorescence signal triggered by the TS and 6 different SNPs when 10 nM Probe-Lock is added to a mixture containing 15 nM candidate strands, 20 nM corresponding reporters, and 20 nM Sink complex. (c) Similar reactions are performed in the presence of 20 nM Proofreader P_snp_. While the TS still shows rapid growth up to about 4 nM in the reported strand concentration, the SNPs show limited increase in the signal even after several days. At least some of the residual activation apparent in (c) is due to a leak reaction between the Probe-lock and the reporter (Figure S61).

We next demonstrate how KP can be incorporated into a simple functional network designed to detect SNPs in a DNA strand where the mutations are anywhere in the displacement domains (Figure 5a). It is imperative to state that we do not propose this particular network as a competitive alternative method to the existing approaches in isolation. Our emphasis is not on the absolute performance of the system before or after proofreading, but on exploring the relative improvement in specificity of a reaction network due to the inclusion of KP. Several DNA strand displacement-based detection methods involving TMSD or toehold exchange have been proposed to differentiate between the wild type and the SNPs.^15,35−38^ Such systems could potentially benefit from adding some form of KP to the detection process.

In the context of SNP detection, it is natural for the strand being tested—either the Target Strand (TS) or SNP-bearing strand (SNP)—to be single stranded. We therefore consider a different reaction network structure relative to the dimerization example while maintaining the central KP functionality. By embedding KP within another functional network, we demonstrate its versatility.

A Probe strand is fully complementary to TS, whereas the SNPs have a mismatch with the Probe at the point of mutation. The candidates (TS or SNPs) bind to the initially blocked Probe via TMSD, providing initial discrimination. This TMSD reveals a second toehold that facilitates the slow formation of a stable complex with fluorescent reporters. Alternatively, proofreader molecules P_snp_ can react with intermediates via the second toehold to form a Probe-P_snp_ complex that is sequestered by a sink S. This proofreading discard pathway provides a second opportunity to discriminate between TS and SNP. We generated a random TS conforming to our design and introduced a single mismatch at different positions of the displacement domain to give six SNP strands (Table S18). We used different nucleotide mismatches to vary the free energy of the mismatched pair and varied the location across the branch migration domain because the effect of mismatches on strand displacement is known to be strongly location-dependent. By design, the same proofreader and probe strands can be used regardless of the specific competing SNP. We triggered the system by adding a blocked Probe to a solution containing the candidate strand, reporter complex, and sink complex (Supplementary Notes 8 and 16). Fluorescent traces with and without 20 nM proofreader are shown in Figure 5; results for other concentrations of proofreader are shown in Figures S61 and S62.

The proofreader improves accuracy in two ways. First, by selectively reacting with the intermediate, the initial rate of product formation for SNPs is reduced by an additional factor of 2–3 relative to TS when proofreaders are used (Figures S63 and S64 and Table S18). Second, the existence of a fuel-consuming discard pathway allows SNP-triggered probes to be consumed (Figure 5c), preventing them from eventually reacting with the reporter, as is typical in the absence of a proofreader strand (Figure 5b). We may once again calculate a discrimination factor D_TS:SNPx_ at the end of the experiment for each proofreader concentration (Supplementary Note 19 and Table S13). In the absence of the proofreader, typical values of *D*_TS:SNPx_ ≈ 1–1.7 are obtained, relative to *D*_TS:SNPx_ ≈ 3–7 for 20 nM of proofreader.

## Conclusions

We demonstrated that kinetic proofreading cycles, a ubiquitous biological motif, can be incorporated in nonenzymatic DNA strand displacement networks. To the best of our knowledge, this work represents the first time that a kinetic proofreading system has been engineered *de novo*. This successful demonstration of KP in a synthetic context sets the groundwork for applying the motif more broadly and opens pathways to explore the fundamental principles of KP in engineerable systems.

We have shown how KP can enhance the discrimination between perfectly matching DNA strands and single nucleotide mutants, both in toeholds and branch migration domains. In this context, the relatively small difference between targets is unavoidable, making it challenging to redesign a system to increase the discrimination at equilibrium between the correct strand and many distinct variants simultaneously. Re-exploiting a single free-energy difference through KP is therefore especially valuable. We note that even if discrimination exists without KP, enhancing that discrimination will always improve detection of low concentrations of a target from within a pool of competitors. KP relies on pushing the template through fuel-driven nonequilibrium cycles. In our design, the large excess of proofreader tends to drive the template through the cycle, although backward steps that reverse the proofreading reaction are not impossible. The presence of downstream reactions, such as the sequestration of waste by reporter molecules, can provide an additional contribution to the thermodynamic drive. Although such a design feature may enhance KP, it is not required for our system—as evidenced by the successful KP in the dimeric production experiments.

Sensitivity to downstream reactions can be a significant factor in responsive circuits. One established approach to effectively discriminate between SNPs is to use toehold exchange probes that are carefully designed so as to render the overall free-energy change of probe-target binding close to zero.^15^ This method ensures that a destabilizing mismatch will have a substantial effect on the yield of products in steady state. These motifs work well in isolation but can struggle to give large discrimination factors in steady state when incorporated into a larger circuit when the downstream reactions will tend to perturb the thermodynamics, as mentioned above. The KP motifs considered here have successfully boosted specificity even when coupled to downstream reactions that bias the thermodynamics and are thus potentially well suited to incorporation into larger functional networks.

In our designs, the free energy used to drive the recognition site through a proofreading cycle comes directly from converting the inputs into waste, rather than coupling to an ancillary fuel molecule as imagined by Hopfield. As a result, rejected monomers are sequestered, rather than being released to attempt incorporation again. In the context of a one-off detection process, as considered here, this absorption of monomers is potentially beneficial. The discard pathway provided by KP allows the incorrect species to be rendered inert, so that incorrect products are never made at all, rather than just delayed as in a scheme powered by ancillary fuel molecules. This suppression of an unwanted signal even on long time scales, as much as the rate advantage provided by KP, will likely aid in the design of more precise synthetic molecular networks. This behavior is distinct from thresholding,^29^ in which a rapid reaction with a thresholding molecule is used to consume inputs so that output signals are only produced when the input concentrations exceed the concentration of the threshold molecule. Mechanistically, KP does not rely on depletion of the proofreader to propagate a signal, and so does not require a minimal input concentration to generate an output. Moreover, thresholding molecules will, if anything, tend to sequester correct species faster than their mutant counterparts.

## Data Availability

All of the fluorescence data as obtained from the plate reader, the Mathematica scripts used to process those data, convert to concentration, and fit them to the ODE models, and the figures are available at 10.5281/zenodo.8132461.

## Supporting Information Available

Detailed experimental procedures, data processing and fitting methods, additional experimental. (PDF)
